# Association of a Hepatopancreas-Specific C-Type Lectin with the Antibacterial Response of *Eriocheir sinensis*

**DOI:** 10.1371/journal.pone.0076132

**Published:** 2013-10-11

**Authors:** Xing-Kun Jin, Xiao-Nv Guo, Shuang Li, Min-Hao Wu, You-Ting Zhu, Ai-Qing Yu, Shang-Jian Tan, Wei-Wei Li, Ping Zhang, Qun Wang

**Affiliations:** School of Life Science, East China Normal University, Shanghai, China; National Cancer Institute, NIH, United States of America

## Abstract

Pattern recognition receptors (PPRs) are part of the initial step of a host defense against pathogens in detecting pathogen-associated molecular patterns. However, determinants of the specificity of this recognition by innate immune molecules of invertebrates remain largely unknown. In this study, we investigated the potential involvement of an invertebrate PRR C-type lectin in the antimicrobial response of the crustacean *Eriocheir sinensis*. Based on the initial expressed sequence tags (EST) of a hepatopancreatic cDNA library, the full-length *EsLecF* cDNA was cloned and determined to contain a 477-bp open reading frame encoding a putative 158-amino-acid protein. A comparison with other reported invertebrate and vertebrate C-type lectin superfamily sequences revealed the presence of a common carbohydrate recognition domain (CRD). *EsLecF* transcripts in *E. sinensis* were mainly detected in the hepatopancreas and were inducible by a lipopolysaccharide (LPS) injection. The recombinant EsLecF (rEsLecF) protein produced via a prokaryotic expression system and affinity chromatography was found to have a wide spectrum of binding activities towards various microorganisms, and its microbial-binding activity was calcium-independent. Moreover, the binding of rEsLecF induced the aggregation of microbial pathogens. Results of the microorganism growth inhibitory assay and antibacterial assay revealed capabilities of rEsLecF in suppressing microorganism growth and directly killing bacteria, respectively. Furthermore, rEsLecF could enhance cellular encapsulation *in vitro*. Collectively, the findings presented here demonstrated the successful isolation of a novel C-type lectin in a crustacean and highlighted its critical role in the innate immunity of an invertebrate.

## Introduction

Invertebrate animals do not have a truly adaptive immunity that is generated by memory and targeted immunoglobulin production as in vertebrates [1], [2]. Nonetheless, invertebrates such as crustaceans are capable of mounting effective innate immune responses to protect against microbial infections [3], [4]. Conserved pathogen-associated molecular patterns (PAMPs), such as lipopolysaccharides (LPS), peptidoglycans and β-1, 3-glucans, which are essential and unique components of virtually all microorganisms but absent in higher organisms [5], can be discriminated by hosts using a wide range of pattern recognition receptors (PRRs) that are highly conserved throughout biological evolution [6] in order to engage a variety of defense mechanisms.

As an important class of non-Toll-like receptor (TLR) PRRs, lectins exist as either transmembrane receptors or soluble proteins in circulating fluids [6], [7]. They play crucial roles in innate immunity such as nonself-recognition and clearance of invading microorganisms [8], [9], via recognition and non-covalent binding of specific sugar moieties to agglutinate pathogens by binding to cell surface glycoproteins and glycoconjugates [10]. C-type lectins are the most diverse and best studied among the lectin superfamily. The term C-type lectin was originally used to distinguish a group of Ca^2+^-dependent (C-type) carbohydrate-binding proteins from the other types of lectins [11]. Members of this large gene family that mediate sugar binding with diverse architecture contain homologous carbohydrate recognition domains (CRDs) which are used to discriminate specific oligosaccharides at cell surfaces and to attach to proteins in the circulation and in the extracellular matrix [12]–[14]. Compared with the well-studied lectins in vertebrates, knowledge of the total innate immune responses mediated by different lectins in invertebrates is still far from complete [15], [16].

In the present study, we investigated the potential involvement of an invertebrate PRR C-type lectin, EsLecF, in the antimicrobial response of the crab *Eriocheir sinensis*. The complete coding region of the *EsLecF* cDNA was obtained by PCR from the initial expressed sequence tags (ESTs) isolated from a hepatopancreatic cDNA library [17]. Expression patterns of *EsLecF* were examined in different tissues of *E. sinensis*, and its immunological response in the hepatopancreas post LPS challenge was also determined. Subsequently, the recombinant EsLecF (rEsLecF) protein was produced via a prokaryotic expression system and affinity chromatography to test its binding activity and antibacterial activity towards different microorganisms, as well as its capacity to induce agglutination and stimulate cellular encapsulation. Our results suggested that the *EsLecF* gene is a constitutively expressed and inducible immune-related gene, and the rEsLecF protein may act as a potent effector in crustacean innate immunity. Thus, these findings provide implications for further research on C-type lectins in invertebrate immunology.

## Materials and Methods

### Animal immune challenge and sample collection

Healthy adult Chinese mitten crabs (n = 200; 80±20 g wet weight) were collected from the Tongchuan Aquatic Product Market in Shanghai, China. After acclimation for one week at 20–25°C in filtered, aerated freshwater, crabs were placed in an ice bath for 1–2 min until lightly anesthetized. Hemolymph was drawn from the hemocoel in the arthrodial membrane of the last pair of walking legs using a syringe (approximately 2.0 ml per crab) with an equal volume of anticoagulant solution (0.1 M glucose, 30 mM citrate, 26 mM citric acid, 0.14 M NaCl, 10 mM EDTA) [18]. The sample was centrifuged at 500× *g* at 4°C to isolate hemocytes. Other harvested tissues (hepatopancreas, gills, muscle, stomach, intestine, heart, testis, ovary, thoracic ganglia and brain) were snap frozen in liquid nitrogen and stored at −80°C prior to nucleic acid analysis. For cloning and expression analysis, tissues of each type harvested from 10 crabs were pooled and ground with a mortar and pestle prior to extraction.

For LPS stimulation, more than 120 crabs were divided equally into two groups (sex ratio 1∶1): each crab of the experimental group was injected with approximately 100 µl of LPS from *Escherichia coli* (Sigma-Aldrich, St. Louis, MO, USA) resuspended in PBS (500 µg/mL) into the arthrodial membrane of the last pair of walking legs, while another control group received 100 µl PBS (pH 7.4). More than five crabs were randomly selected at each time interval of 0 (as blank control), 4, 8, 12, 24, 48 and 72 h post LPS injection. The hepatopancreas was harvested from each crab as described above and stored at −80°C after the addition of 1 mL Trizol reagent (Invitrogen, Carlsbad, CA, USA) for subsequent RNA extraction.

### Total RNA extraction and first-strand cDNA synthesis

Total RNA was extracted from *E. sinensis* tissues using Trizol^®^ reagent (RNA Extraction Kit, Invitrogen) under the guidance of the manufacturer's protocol. The total RNA concentration and quality were estimated using spectrophotometry at an absorbance at 260 nm and agarose-gel electrophoresis, respectively.

Total RNA (5 μg) isolated from the hepatopancreas was reverse transcribed using the SMARTer™ RACE cDNA Amplification kit (Clontech, Mountain View, CA, USA) for full-length cDNA cloning. For quantitative real-time RT-PCR (qRT-PCR) expression analysis, total RNA (4 μg) was reverse transcribed using the PrimeScript™ Real-time PCR Kit (TaKaRa, Shiga, Japan).

### Cloning of full-length EsLecF cDNA

The C-type lectin *EsLecF* partial cDNA sequence was extended using 5′ and 3′ RACE (SMARTer™ RACE cDNA Amplification kit, Clontech), and gene-specific primers (Table 1) were designed based on the original ESTs isolated from a hepatopancreatic cDNA library [17]. The 3′ RACE PCR reaction was carried out in a total volume of 50 μl containing 2.5 μl (800 ng/μl) of the first-strand cDNA reaction as a template, 5 μl of 10X Advantage 2 PCR buffer, 1 μl of 10 mM dNTPs, 5 μl (10 μM) of gene-specific primer (Table 1), 1 μl of Universal Primer A Mix (UPM; Clontech), 34.5 μl of sterile deionized water (RNase free, TaKaRa, Japan), and 1 U of the 50× Advantage 2 polymerase mix (Clontech). For the 5′ RACE, UPM was used as forward primers in PCR reactions in conjunction with the reverse gene-specific primers (Table 1). PCR amplification conditions for both the 3′ and 5′ RACE were as follows: 5 cycles at 94°C for 30 s, 72°C for 3 min; 5 cycles at 94°C for 30 s, 70°C for 30 s, and 72°C for 3 min; 20 cycles at 94°C for 30 s, 68°C for 30 s, and 72°C for 3 min. PCR amplicons were size separated and visualized on an ethidium bromide stained 1.2% agarose gel. Amplicons of expected sizes were purified with the Wizard^®^ SV Gel and PCR Clean-Up System (Promega, Madison, WI, USA), inserted into a pZeroBack/Blunt vector (Tiangen, Shanghai, China) and transformed into TOP10 *E. coli*. Positive clones containing inserts of an expected size were two-way sequenced using 23-mer and 24-mer primers (Table 1).

**Table 1:** Primer sequences.

Primer application	Sequence (5′–3′)
***EsLecF full-length cloning***	
5′RACE	ACCCGCTGGCTGGTCGTTGATGT
3′RACE	CTTCTTGACAGCAGCACACACCCTC
***Quantitative RT-PCR***	
*EsLecF*-F	CTCTATTTCGTGAAGTCCAAGGC
*EsLecF*-R	GGTGCCCAAGGGTATTTCTGTA
*β-actin*-F	CTCCTGCTTGCTGATCCACATC
*β-actin*-R	GCATCCACGAGACCACTTACA
***Coding region cloning***	
rEsLecF-F	CCGGAATTCCAATGTCCTGCTGCTTTTGTAG
rEsLecF-R	CCCAAGCTTTTATTAGATTCGACAGATGCCCATA
***Sequencing***	
23-mer	CGACTCACTATAGGGAGAGCGGC
24-mer	AAGAACATCGATTTTCCATGGCAG
T7 promoter	TAATACGACTCACTATAGG
T7 terminator	CACCGCTGAGCAATAACTAGC

### Transcription analysis by qRT-PCR

Quantitative RT-PCR was performed using SYBR^®^ Premix ExTaq^TM^ (TaKaRa) with a *EsLecF* gene-specific primer pair (Table 1) producing a 231-bp amplicon. PCR was carried out in a CFX96 instrument (Bio-Rad, Hercules, CA, USA) as follows: 30 cycles at 94°C for 30 s, 58°C for 30 s, and 72°C for 1 min. Internal control PCR reactions for *β-actin* were performed in a separate tube, as described above with the exception of an alternative gene-specific primer pair (Table 1), which was designed based upon a cloned *E. sinensis β-actin* cDNA fragment to produce a 276-bp amplicon. All qRT-PCR experiments were completed in triplicate using independently extracted RNA. Both for the tissue transcription assay and the immune challenge transcription assay, PCR templates were obtained as above. *EsLecF* relative expression levels were calculated by the 2^−ΔΔCt^ comparative *C_T_* method [19].

### Construction of recombinant expression plasmids

The coding region of *EsLecF* was amplified from the hepatopancreas cDNA template using specific primer pairs designed with *Bam*HI and *Hin*dIII endonuclease sites included at the 5′ end of the forward and reverse primers, respectively (Table 1). The PCR products were double digested with the same endonucleases (New England Biolabs, Ipswich, MA, USA), purified using the Wizard® SV Gel and PCR Clean-Up System (Promega) and ligated to the corresponding cohesive ends of the pET32a plasmid (Novagen, Darmstadt, Germany) with T4 ligase (New England Biolabs). The cloned *EsLecF* insert was confirmed by two-way DNA sequencing with T7 primer pairs (Table 1).

### Expression and purification of rEsLecF protein

The positive recombinant plasmids were transformed into *E. coli* BL21-DE3 (Tiangen, China) to express recombinant proteins. The empty pET32a plasmid which expresses the recombinant thioredoxin protein (rTrx) was used as a blank control. After extensive culture until the logarithmic phase (OD_600_ value reaching 0.6) in ampicillin-containing Luria-Bertani (LB) broth, isopropyl-b-D-1-thiogalactopyranoside (IPTG) was added to the medium (1 mM final concentration) to induce the rEsLecF protein expression. After aerobic culture overnight at 37°C, the bacteria were collected by centrifugation (6000× *g*). The pellets were resuspended in 8 ml of Guanidine Lysis Buffer and ultrasonicated. The 8 ml lysate was then added to a prepared affinity purification column using His-binding resin chromatography (Invitrogen) following the manufacturer's instructions under hybrid conditions. The resin was kept suspended in the lysate solution for 30–60 min to allow the target protein with a His-tag to bind to the resin of the purification column. After centrifugation at low speed (800× *g*) and washing the resin with 8 ml of Native Wash Buffer four times, the protein was finally eluted with 8–12 ml of Native Elution Buffer. Routine protein estimation was conducted using the Bradford method with bovine serum albumin (Bio-Rad, Hercules, CA, USA) as the standard.

### Western blotting assays

A set of Western blotting assays was designed to determine the binding activity of the rEsLecF protein to various microorganisms, as well as the requirement of calcium for this binding activity. Three Gram-positive bacteria (*Staphyloccocus aureus*, *Bacillus subtilis* and *Microbacterium lactium*), three Gram-negative bacteria (*Vibrio parahemolyticus*, *Aeromonas hydrophila* and *E. coli*) and one yeast strain (*Pichia pastoris*) were extensively cultured in the appropriate medium (LB for bacteria, Yeast Peptone Dextrose YPD for yeast) and then collected by centrifugation (6000× *g*), followed by resuspension in 2 ml TBS (50 mM Tris-HCl, 150 mM NaCl, pH 7.5; OD_600_ ≈1.0). The microorganisms (2×10^7^ cells/ml in 500 μl TBS) were incubated with purified rEsLecF protein (0.4 mg/ml; 500 μl) for 1 h at 37°C. Thereafter, the microorganisms were mixed, pelleted (6000× *g*, 5 min), washed four times with TBS and then eluted with 10% SDS. Eluents were suspended in SDS-PAGE loading buffer and separated by 12% SDS-PAGE and transferred to a PVDF membrane. The membrane was blocked with 5% dry skim milk in TBS (20 mM Tris–HCl, 150 mM NaCl, pH 8.0) at room temperature for 2 h and then subjected to immunoblot assays by incubating at 4°C overnight with an anti-His-tag mouse antibody (Cwbio, Shanghai, China) (1∶500) diluted with 2% dry skim milk in TBS (20 mM Tris–HCl, 150 mM NaCl, pH 8.0). Positive reactivity was detected with a horseradish peroxidase-conjugated goat anti-mouse IgG (1∶2000) (Cwbio) and visualized using the ChemiDoc XRS imaging system (Bio-Rad).

### Microorganism agglutination activity assays

The microorganism agglutination activity of rEsLecF protein was assessed as previously described [20]. The Gram-positive bacteria *S. aureus*, Gram-negative bacteria *E. coli* and the yeast *P. pastoris* were cultivated overnight and collected by centrifugation (6000× *g*). The microbial pellets were washed, resuspended in TBS (50 mM Tris-HCl, 150 mM NaCl, pH 7.5) and adjusted to an OD_600_ value of approximately 1.0. The pelletted microorganisms (10 μl) were incubated with rEsLecF protein (50 μl) in the presence or absence of 10 mM CaCl_2_. TBS solution (50 μl) was used as a control. The mixtures were incubated at room temperature (1–2 h), and reactions were observed by bright light microscopy (Leica, DM4000B, Wetzlar, Germany).

### Microorganism growth inhibition assays

The Gram-positive bacteria *S. aureus*, Gram-negative bacteria *E. coli* and the yeast *P. pastoris* were chosen to test the microorganism growth inhibitory activity of the rEsLecF protein. Single colonies of the chosen strains were selected and cultured at 30°C in 1 ml LB or YPD broth as appropriate, and the rEsLecF protein was added at three final concentrations (0, 20 and 200 μg/ml). Each sample was incubated with aeration at 200 rpm, and the OD_600_ was measured every 2 h. Growth curves of the microorganisms were drawn based on data from three independent experiments.

### Antibacterial activity assays

Antimicrobial activities of rEsLecF protein against *E. coli* and *S. aureus* also were assessed on agar petri dishes. Selected single colonies were intensively cultured in LB medium and subsequently collected by centrifugation (6000× *g*) for 10 min and resuspension in TBS (50 mM Tris-HCl, 150 mM NaCl, pH 7.5). Each of the prepared microbes was smeared on a 100 mm×20 mm agar petri dish. Perforex was used to produce 10-mm diameter pores. Equal concentrations of either the antibiotic ampicillin, rTrx or rEsLecF proteins (50 μg/100 μl TBS) were added into the pores, and then the plates were incubated at 37°C for 16 h (*E. coli*) or 30°C for 16 h (*S. aureus*). TBS (100 μl) was added as a blank control.

### Hemocyte encapsulation assay

Hemocyte encapsulation assays with the rEsLecF protein were performed as previously described [20]. In this study, Ni-NTA agarose beads (Novagen) were washed three times, equilibrated in TBS (50 mM Tris-HCl, 150 mM NaCl, pH 7.5) and subsequently incubated with His-tagged rEsLecF protein at room temperature for 1 h. Protein-coated beads were washed with TBS four times and resuspended in TBS. Purified hemocytes were added to each group of the protein-coated beads and incubated at room temperature for 6 or 24 h. The final reactions were observed by light microscopy (Leica, DM4000B). The control was rTrx protein.

### Bioinformatics analysis

Similarity analysis was performed with BlastX (http://www.ncbi.nlm.nih.gov/), and multiple sequence alignments of CRD domains from *E.sinensis* C-type lectins were conducted using ClustalW2 and derived using WebLogos (http://weblogo.berkeley.edu/). Signal sequence and motif predictions were performed using SMART (http://smart.embl-heidelberg.de/). An unrooted Maximum Likelihood phylogenetic tree of C-type lectins amino acids sequences was constructed with MEGA5.0. Protein molecular weights were predicted by ProtScale (http://web.expasy.org/protscale/).

### Statistical analysis

Statistical analysis was performed using SPSS software (Ver11.0). The data are presented as the mean ± standard error (S.E.). Statistical significance was determined by one-way ANOVA and *post hoc* Duncan multiple range tests. Significance was set at *P*<0.05.

## Results

### Characterization of EsLecF full-length cDNA

**Figure 1:**
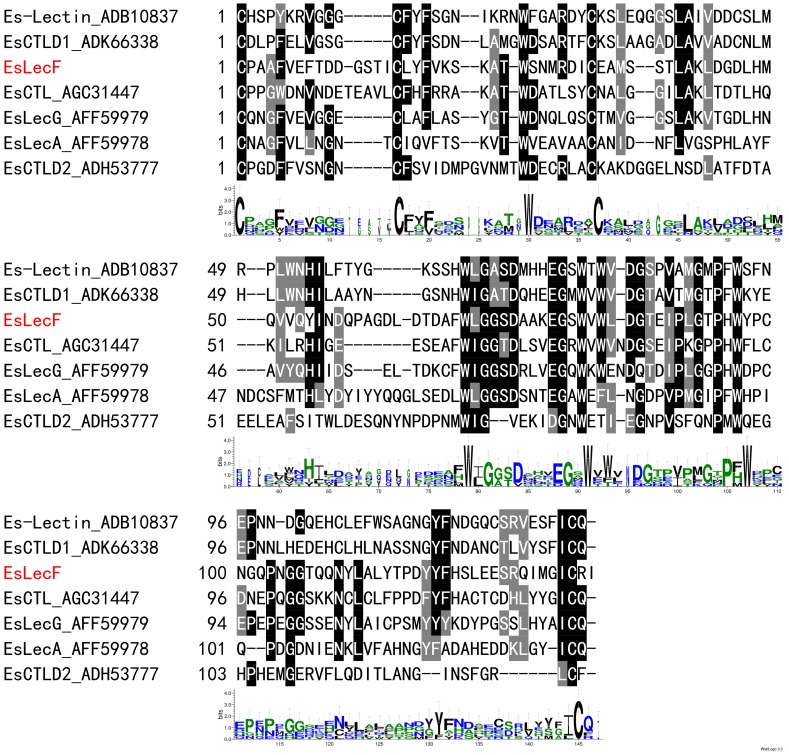
Multiple amino acid sequences alignment of carbohydrate recognition domains (CRD) among *EsLecF* and the other *E.sinensis* C-type lectin isoforms by ClustalW2. The consensus amino acid sequences of the CRD in different *E.sinensis* isoforms were derived using WebLogos.

The obtained full-length cDNA of the *E. sinensis* C-type lectin, designated as *EsLecF* (GenBank accession number JX129178), was 873 bp and contained a 477-bp ORF encoding a 158-amino-acid protein, as well as a 176-bp 5′ UTR and a 220-bp 3′ UTR. The deduced amino acid sequence of *EsLecF*, as analyzed using the SMART program, was detected to have a typical domain architecture the same with other C-type lectins, including a signal peptide and one CRD with a QPN (Gln-Pro-Asn) motif (Fig. 1 and Fig. S1).

### Phylogenetic analysis of EsLecF

A maximum likelihood phylogenetic tree was produced based on analysis of the EsLecF deduced amino acid sequence with representative invertebrate and vertebrate homologs. The lectin tree contained two distinct clades distinguishing invertebrates (crustaceans) from vertebrates. EsLecF and six other *E. sinensis* lectins (deep red branch lines) occurred in the same clade of invertebrates but were separated into distinct subclades (Fig. S2).

### Transcription patterns of EsLecF

**Figure 2:**
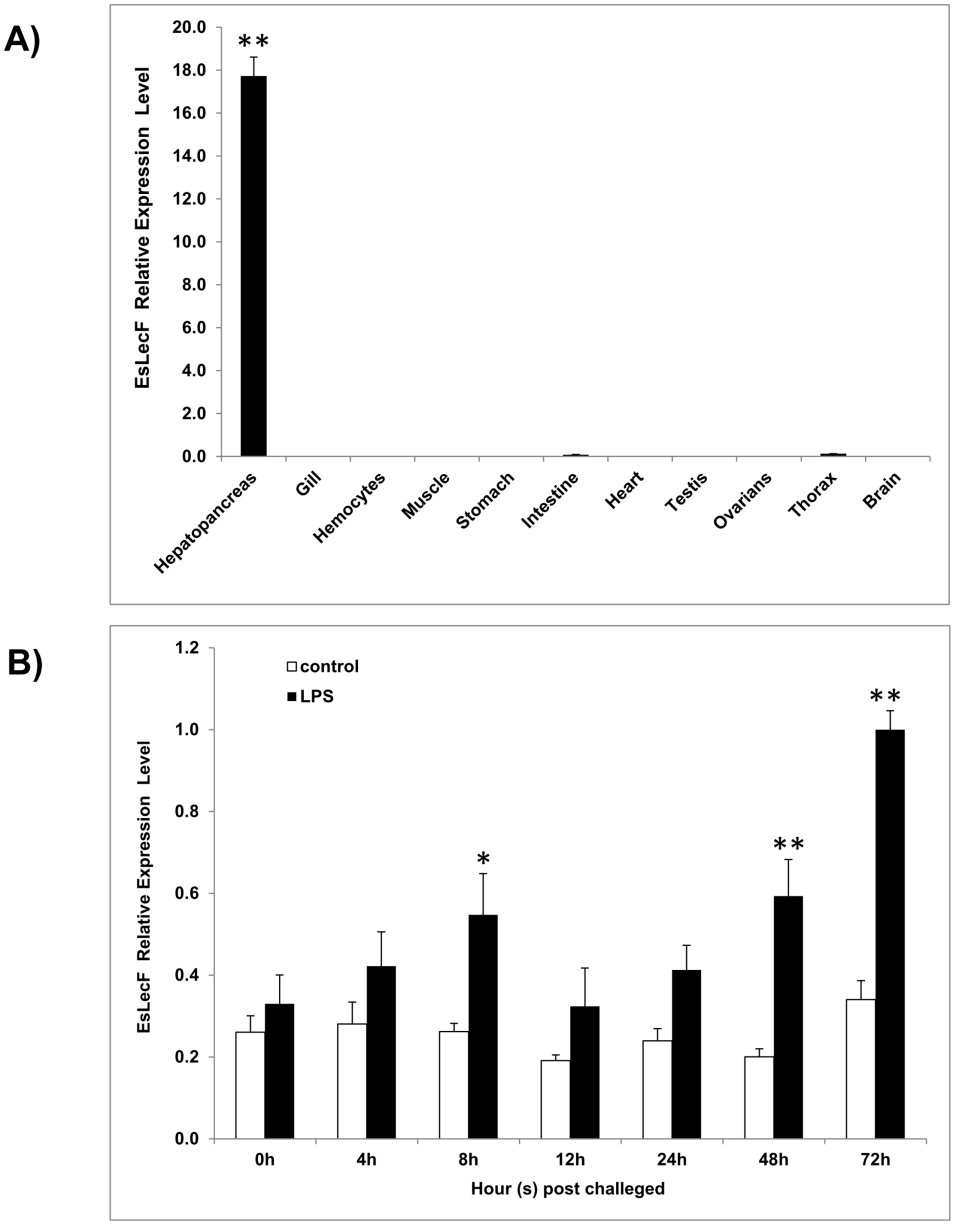
Temporal mRNA expressions of *EsLecF* in various tissues (black bars) (A) or in the hepatopancreas in response to LPS challenge (B). *EsLecF* mRNA expression relative to β-actin was compared between hepatopancreas samples collected from crabs injected with LPS (black bars) or vehicle control (white bars). Bars represent means ± S.E. (n = 6). *P<0.05; **P<0.01.

As determined by qRT-PCR, the *EsLecF* transcript was observed at an abundant level in the hepatopancreas, an immune organ, while it was rarely found in the intestine and thorax (Fig. 2A). At 8 h and 48 h after injection with LPS, significantly higher levels *EsLecF* than the control were detected (*P*<0.05), peaking up to 3 times above the blank control after 72 h (Fig. 2B).

### Expression and purification of rEsLecF protein

**Figure 3:**
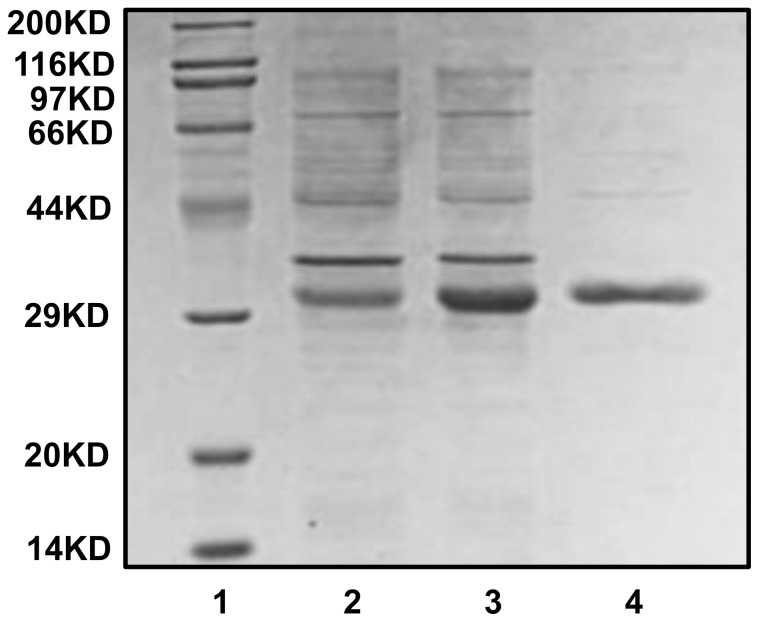
Staining of expressed and purified rEsLecF protein with Coomassie Brilliant Blue. Lane 1, protein marker; lane 2, lysate of *E. coli* transformed with pET32a-*EsLecF* without induction; lane 3, lysate of *E. coli* transformed with pET32a-*EsLecF* after induction by IPTG; lane 4, rEsLecF protein after affinity chromatography purification with His-tag binding resin.

The deduced protein of *EsLecF* was predicted to have a molecular weight of approximately 15.3 kDa without the signal peptide. In the prokaryotic expression system, rEsLecF was expressed as a fusion protein with the additional His-tag and Trx-tag at the N-terminus, which increased the molecular mass of the expressed protein to approximately 32.6 kDa. The coding frames were confirmed by two-way sequencing of recombinant plasmids, and the purified recombinant proteins were confirmed by SDS-PAGE electrophoresis and visualization by Coomassie Brilliant Blue staining (Fig. 3).

### Microorganism binding activities

**Figure 4:**
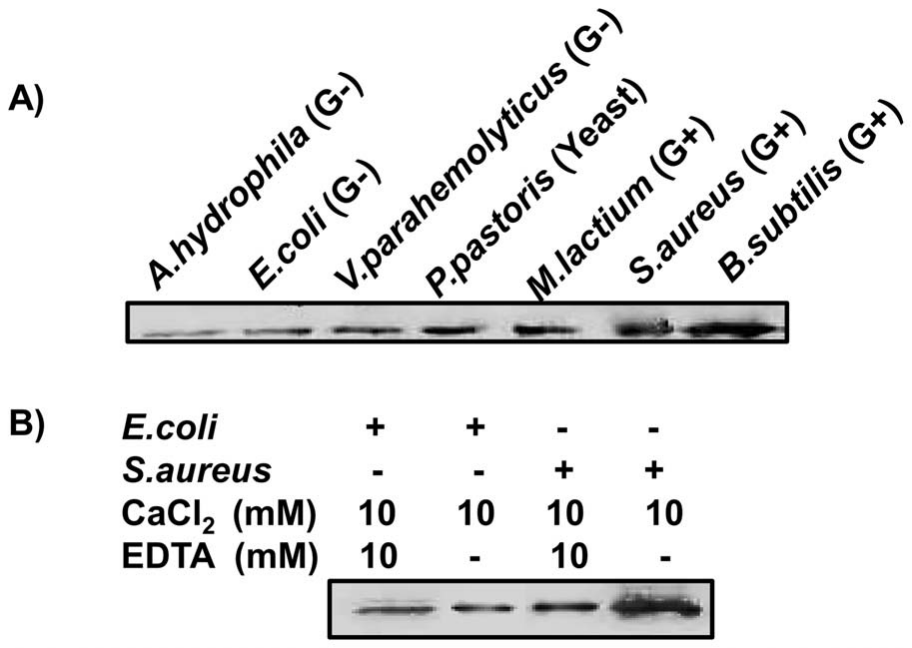
Microorganism binding assays of rEsLecF protein. (A) Logarithmic growth phase microbial strains were monitored after incubation with rEsLecF protein. At indicated times, the microbial samples were ultrasonicated, and the washed pellets were subjected to SDS-PAGE and Western blot analysis with an anti-His-tag antibody. B) The calcium for rEsLecF protein-microbial binding was not essential. The presence of calcium could dramatically increase the affinity of rEsLecF protein binding to microorganisms.

The rEsLecF protein was capable of binding with almost all tested microorganisms, especially to yeast and Gram-positive bacteria, while it exhibited comparatively weaker binding activities towards Gram-negative bacteria (Fig. 4A). In the absence of calcium (removed by addition of 10 mM EDTA), rEsLecF could still bind to the Gram-positive bacteria *S. aureus* and the Gram-negative bacteria *E. coli*, suggesting that the rEsLecF protein bound to the microbes in a calcium-independent way. In addition, the presence of calcium could dramatically increase the affinity of rEsLecF protein binding to the microorganisms (Fig. 4B).

### Microorganism agglutination by rEsLecF

**Figure 5:**
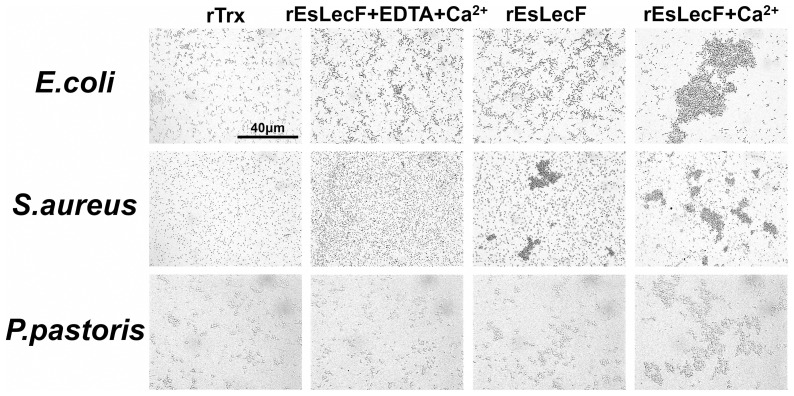
Microorganism agglutination assays of rEsLecF protein in the absence or presence of calcium. *E. coli*, *S. aureus* and *P. pastoris* were incubated with rEsLecF and either (1) EDTA + Ca^2+^, (2) TBS as blank or (3) Ca^2+^. Scale bar: 40 μm. rTrx protein was set as negative control.

To determine whether the binding activity of rEsLecF could induce the aggregation of microbial pathogens, the recombinant protein was incubated with Gram-positive bacteria *S. aureus*, Gram-negative bacteria *E. coli* and the yeast *P. pastoris*. Observations by light microscopy indicated that rEsLecF was capable of inducing aggregation of all three types of microbes tested. In the absence of calcium (removed by addition of EDTA), the *S. aureus* aggregations induced by rEsLecF protein were completely inhibited, while those of *E. coli* and *P. pastoris* were not. In the presence of calcium, all microbial aggregations induced by the rEsLecF protein were consistently enhanced (Fig. 5).

### Microorganism growth inhibitory and antibacterial effects of rEsLecF

**Figure 6:**
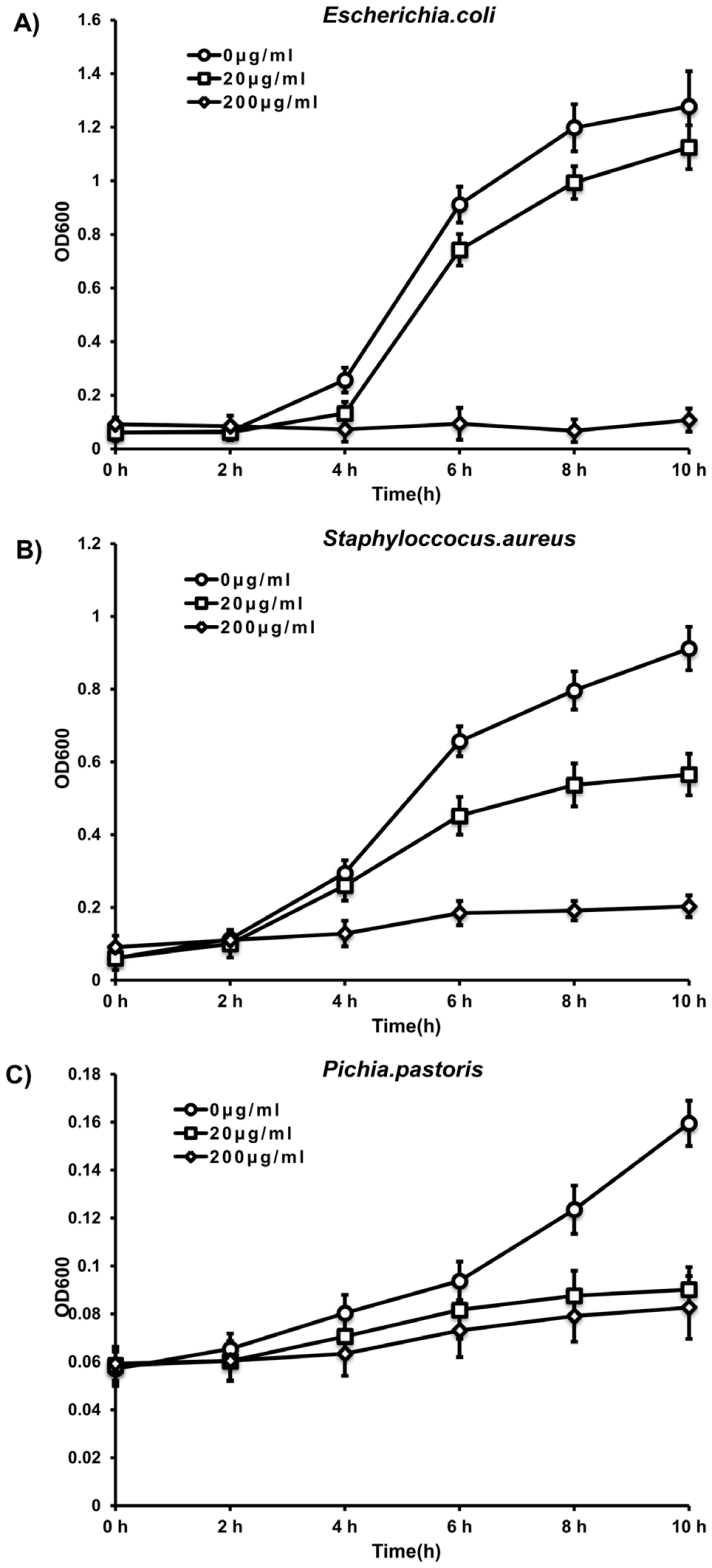
Microorganism growth inhibition assays with rEsLecF protein. *S. aureus*, *E. coli* and *P. pastoris* were mixed with 0, 20 or 200 μg/ml of rEsLecF protein, and OD_600_ measurements were taken at different growth time intervals. Bacterial growth curves of *E. coli* (A), *S. aureus* (B) and *P. pastoris* (C), each incubated with rEsLecF, are shown.

To determine the antimicrobial activity of rEsLecF, its inhibitory effect on the growth of microorganisms was examined. The rEsLecF protein could significantly inhibit the growth of all tested microbe strains compared with the TBS control (rEsLecF, 0 μg/ml). Furthermore, the antimicrobial effects were dose-dependent, with the growth slightly suppressed by rEsLecF at the concentration of 20 μg/ml and more severely inhibited when increased to 200 μg/ml (Fig. 6).

**Figure 7:**
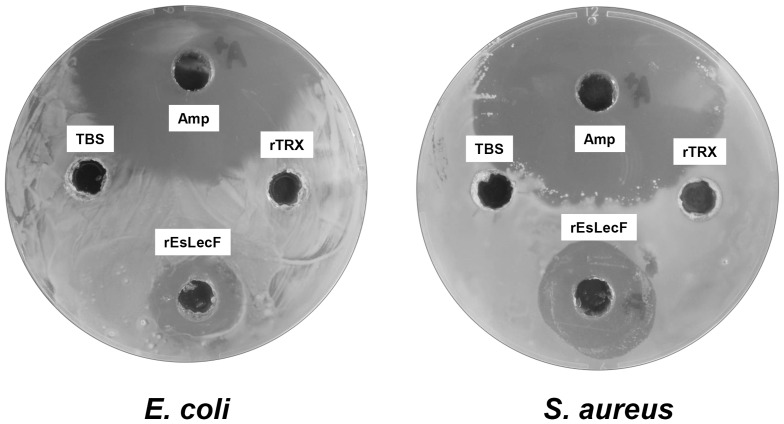
Antibacterial activities of rEsLecF protein against *E. coli* and *S. aureus* on petri dishes. TBS (100 μl) alone (blank) or with ampicillin (Amp, 50 μg) or rTrx or rEsLecF protein was added to each pore of the agar plates and mixed with microbes. The plates were incubated at 37°C for 16 h (*E. coli*) or 30°C for 16 h (*S. aureus*) before observing the transparent ring around the pores, which indicate antibacterial activity.

The antibacterial activity of rEsLecF protein was also detected on agar petri dishes. Transparent rings were found around the pores with rEsLecF protein and ampicillin added, indicating that the microbial killing effects of rEsLecF were analogous to those of the antibiotic against *E. coli* and *S. aureus*, compared with the TBS and rTrx control (Fig. 7).

### In vitro cellular encapsulation assay

**Figure 8:**
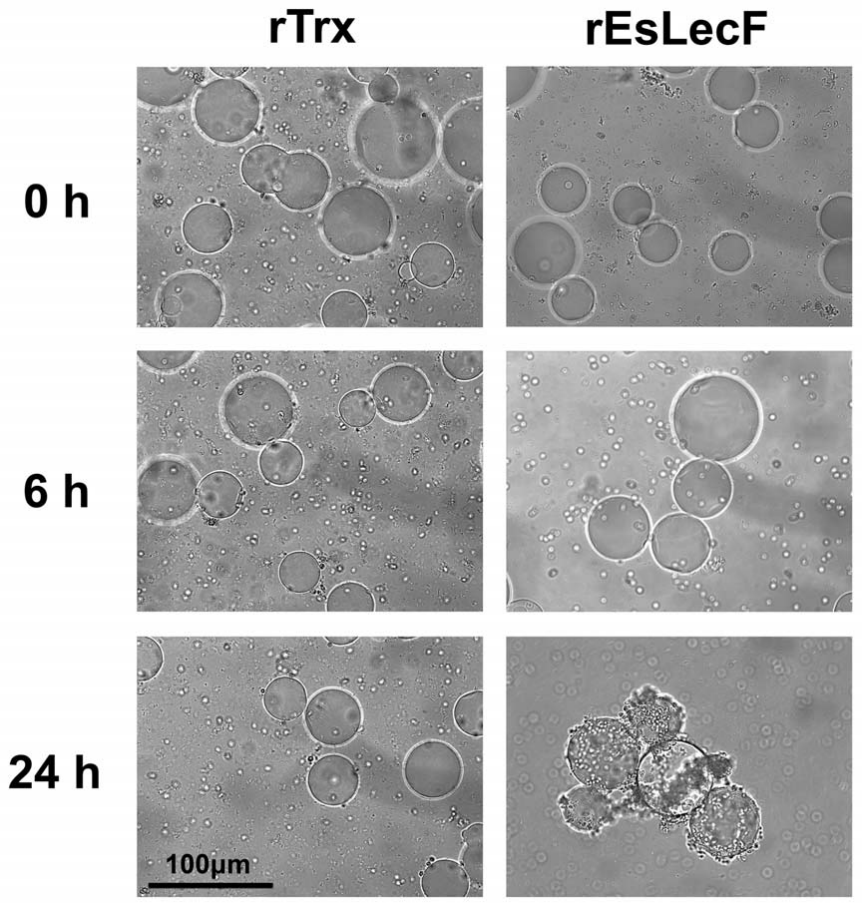
Recombinant EsLecF protein promotes hemocyte encapsulation *in vitro*. Nickel agarose beads coated with rTrx or rEsLecF were incubated with hemocytes from *E. sinensis*. The beads were observed by microscopy after 0, 6 and 24 h of incubation. Scale bar: 100 μm.

The potential ability of rEsLecF protein to promote cellular encapsulation of microorganisms *in vitro* was carried out by incubating nickel agarose beads coated with rEsLecF or rTrx with hemocytes from *E. sinensis.* Subsequent observations indicated that the agarose beads coated with rEsLecF could strongly stimulate the encapsulation of hemocytes up to 24 h of incubation, compared with those coated with rTrx protein (Fig. 8).

## Discussion

Lectins are functional as significant pattern recognition proteins in innate immunity via nonself-recognition and clearance of invading microorganisms [7]–[9]. Among them, C-type lectins form a large gene family with multiple functions participating in cell adhesion, endocytosis, pathogen neutralization, glycoprotein clearance and phagocytosis [21]–[23]. In invertebrates, lectins have been reported to contribute in innate immune responses, such as prophenoloxidase activation [24]–[26], enhancement of encapsulation [16], [27], [28], nodulation of hemocytes [9], [29], opsonization [30], [31] and antimicrobial activity [32], [33]. However, compared to the available knowledge of vertebrate lectins, the molecular features and functions of lectins in crustaceans are just beginning to be understood [16].

Although alignment of the deduced amino acid sequence of the novel C-type lectin EsLecF cloned from the *E. sinensis* hepatopancreas in this study showed a relatively conserved CRD, variations in some motifs were found. For instance EsLecF contains a unique “QPN” (Gln-Pro-Asn) motif, instead of the typical “QPD” or “EPN” motif which had been predicted to be ligand-binding specific for galactose or mannose, respectively [14] (Fig. 1 and Fig. S1). This domain contains a characteristic double-loop stabilized by highly conserved disulfide bridges, Ca^2^-binding sites and carbohydrate-binding sites [14]. Along with the other six *E. sinensis* lectins, *EsLecF* was grouped in the same invertebrate clade, but distinct subclades, in the maximum likelihood phylogenetic tree (Fig. S2). This result suggests that these lectins must play diverse roles in crab immunity. As determined by qRT-PCR, the *EsLecF* transcript was mainly observed in the hepatopancreas (Fig. 2A). This result was consistent with the hepatopancreas-specific expression patterns of C-type lectins in most shrimps [34]–[37], but it was quite different with the other widely distributed C-type lectins in *E. sinensis* [38]–[41]. The lack of dual- or multi-CRDs, such as that found in shrimps, of C-type lectins in crabs suggests that they must function in different manners. Importantly, the *EsLecF* transcript was significantly induced post LPS stimulation in the hepatopancreas (Fig. 2B). Diverse crustacean C-type lectins with structural and functional variations are mainly expressed in the hepatopancreas and constitute a pathogen-recognition network against invading bacteria and viruses [3]. In the shrimp *Litopenaeus vannamei*, LvCTL1 was found to be highly induced in hepatopancreas at 24, 36 and 48 h after an injection with white spot syndrome virus (WSSV) [34]. In another study of the crab *E. sinensis*, both EsCTLDcp-1 and EsCTLDcp-2 were shown to be upregulated in the hepatopancreas after LPS challenge [41]. During an acute immune response in crabs, gene transcription can be regulated by various external and internal factors. In this study, the significant upregulation in expression of *EsLecF* in the hepatopancreas after 8, 48 and 72 h after stimulation with LPS possibly occurred to produce additional mature functional proteins to recognize the PAMPs.

Lectins are capable of recognizing and non-covalently binding to specific saccharide moieties and therefore agglutinate cells by binding to cell surface glycoproteins and glycoconjugates [10], [16]. In our study, the recombinant EsLecF protein was found to be capable of binding a wide range of tested microorganisms, especially Gram-positive bacteria and yeast (Fig. 4A). The key feature distinguishing C-type lectins from the other types of lectins is a calcium dependence of the carbohydrate binding process [11]. In the absence of calcium, rEsLecF could still bind to Gram-positive bacteria *S. aureus* and Gram-negative bacteria *E. coli*, indicating that the calcium was not essential for rEsLecF protein binding to microbes. In additon, the presence of calcium could significantly enhance the binding activities of rEsLecF protein towards microorganisms (Fig. 4B). In the microbial aggregation assay, rEsLecF was capable of inducing aggregations of *E. coli*, *S. aureus* and *P. pastoris*. Interestingly, aggregations of *S. aureus* and *P. pastoris*, but not that of *E. coli*, triggered by rEsLecF were completely inhibited in the absence of calcium. By contrast, all microbial aggregations induced by the rEsLecF protein were dramatically enhanced in the presence of calcium (Fig. 5). Although the C-type lectin family members were initially characterized by their calcium dependence in ligand binding, many other C-type lectins have exhibited the same calcium-dependent or -independent features. In the worm *Manduca sexta*, immulectin was shown to induce the agglutination of *S. aureus* in a calcium-dependent manner [24]; however, immulectin-2 does not require calcium for its binding activity [42]. In the shrimp *Fenneropenaeus chinensis*, the mature Fc-hsL protein requires calcium for its agglutinating activity, but not for microorganism binding or antimicrobial activity [43]. In the amphioxus *Branchiostoma belcheri*, calcium is essential in the hemagglutination and microbial aggregation but not in the microbial binding and growth suppression mediated by AmphiCTL1. Although it is not a direct ligand or essential factor for binding, AmphiCTL1 may affect the formation of dimers or oligomers, which are required for agglutinating activities [32]. In our previous study, the rEsLecA and rEsLecG proteins were determined to could bind with microbial pathogens in a calcium-independent way [44]. In this study, it seems likely that both the microbial-binding activities and the microbial-aggregating activities of rEsLecF protein is calcium-independent, in addition, they could be intensively enhanced by the calcium.

Innate immunity involves a series of PRRs which exert antimicrobial activity in addition to immune recognition. These PRRs play crucial roles in nonspecific host defenses by preventing or inhibiting pathogenic invasion via specifically recognizing potential PAMPs [5], [6]. As important PRRs, many C-type lectins are reported to have antibacterial activities. For instance, in the tunicate *Polyandrocarpa misakiensis*, a calcium-dependent galactose-binding lectin was shown to exhibit potent antibacterial activity [45]. In the amphioxus *B. belcheri*, AmphiCTL1 has demonstrated the ability to recognize a wide range of microorganisms and to directly kill microbes via interaction with peptidoglycan and glucan, representing a new function for invertebrate lectin-mediated immunity [32]. In the shrimp *F. chinensis*, one C-type lectin Fc-hsL has displayed remarkable antimicrobial activity, with especially high activity against Gram-positive bacteria and some fungi and moderate activity against Gram-negative bacteria [43]. Moreover, another C-type lectin FcLec4 was demonstrated to directly facilitate the clearance of *Vibrio anguillarum in vivo* [46]. In our study, the rEsLecF protein was determined to have obvious inhibitory effects on the growth of all tested microbial strains of *S. aureus*, *E. coli* and *P. pastoris* compared with the TBS control (rEsLecF, 0 μg/ml). Furthermore, the antimicrobial activity of the rEsLecF protein was dose-dependent, as the growth of organisms was still maintained with 20 μg/ml rEsLecF but nearly completely inhibited by 200 μg/ml rEsLecF (Fig. 6). Furthermore, the antibacterial activities of rEsLecF on petri dishes were comparable to that of ampicillin against *E. coli* and *S. aureus* (Fig. 7). The rEsLecF protein likely is involved in the immobilization of bacteria, binding and destruction of bacteria cells walls, which would lead to inhibition or termination of microorganism growth [32], [33]. Nonetheless, further investigation should be performed in order to clarify the molecular mechanisms underlying this antibacterial activity.

Crustaceans have an incompletely closed vascular system, where hemocytes participate in the defense against intruding pathogens in addition to functioning in oxygen transport [47], [48]. In the crustacean innate immune system, hemocytes not only can synthesize and exocytose a battery of bioactive molecules but also mediate rapid immune reactions such as coagulation and encapsulation [16], [49]. Encapsulation is a cellular immune response that solely exists in invertebrates to fight against foreign particles too large for phagocytosis by individual hemocytes. Unlike phagocytosis, encapsulation results in a mutilayered, overlapping sheath of hemocytes around the invader, which is ultimately eliminated within the capsule [31]. With a broad range of biological functions, C-type lectins have been reported to promote cellular encapsulation in invertebrates [10], [16]. In the worm *M. sexta*, both IML-1 and IML-3 can initiate attachment of hemocytes to foreign objects, hence stimulating encapsulation but not melanization [27], [28], whereas IML-2 not only can enhance encapsulation but also lead to melanization [8], [28]. Coating of agarose beads with recombinant DL2 and DL3 of the fly *Drosophila melanogaster* has been shown to enhance their encapsulation and melanization by hemocytes *in vitro* [50]. Sepharose 4B beads coated with recombinant LvCTLD were found to be encapsulated by shrimp (*L. vannamei*) hemocytes followed by melanization by 24 h post-encapsulation [51]. Likewise, in the other studies of the crab *E. sinensis*, recombinant EsLecA, EsLecG and EsCTL proteins could promote hemocyte encapsulation *in vitro* [38], [44]. In the current study, Ni-NTA agarose beads coated with rEsLecF could strongly induce the encapsulation of hemocytes up to 24 h of incubation, compared with those coated with rTrx protein (Fig. 8). These results indicated that rEsLecF could facilitate the recognition of invading foreign particles by hemocytes, subsequently initiating their encapsulation, which is consistent with the abilities of other invertebrate C-type lectins reported [27], [28], [38].

## Conclusions

The present study described the successful cloning, sequence analysis, tissue-specific distribution and immune responsiveness of a novel C-type lectin from *E. sinensis*. The rEsLecF protein was obtained and characterized with microbial binding and direct killing activities. Furthermore, the rEsLecF protein could stimulate cellular encapsulation *in vitro*. Future investigations should be directed towards understanding the specific mechanisms of interactions of C-type lectins with various PAMPs, as well as their antibacterial activities *in vivo*.

## Supporting Information

Figure S1 **Nucleotide and deduced amino acid sequences of** ***EsLecF*** **.** The nucleotide sequence is numbered from the first base at the 5′ end. The first methionine (M) is numbered as the first deduced amino acid. The bold underline indicates the location of the signal peptide (1–21 aa). The CRD is shaded (23–158 aa). The functional motif of “QPN” is bolded.
(TIF)

Figure S2 **Unrooted maximum likelihood phylogenetic tree of** ***EsLecF*** **(labeled with a square).** Amino acid sequences of C-type lectins obtained from a BlastP homology search show high similarities. The branches of *E. sinensis* lectins are marked in deep red color.
(TIF)
