# Supplementary figures and images for: Association of a Hepatopancreas-Specific C-Type Lectin with the Antibacterial Response of Eriocheir sinensis
Source: PLoS One. 2013 Oct 11;8(10):e76132. doi: 10.1371/journal.pone.0076132 (PMC3795701; doi:10.1371/journal.pone.0076132)

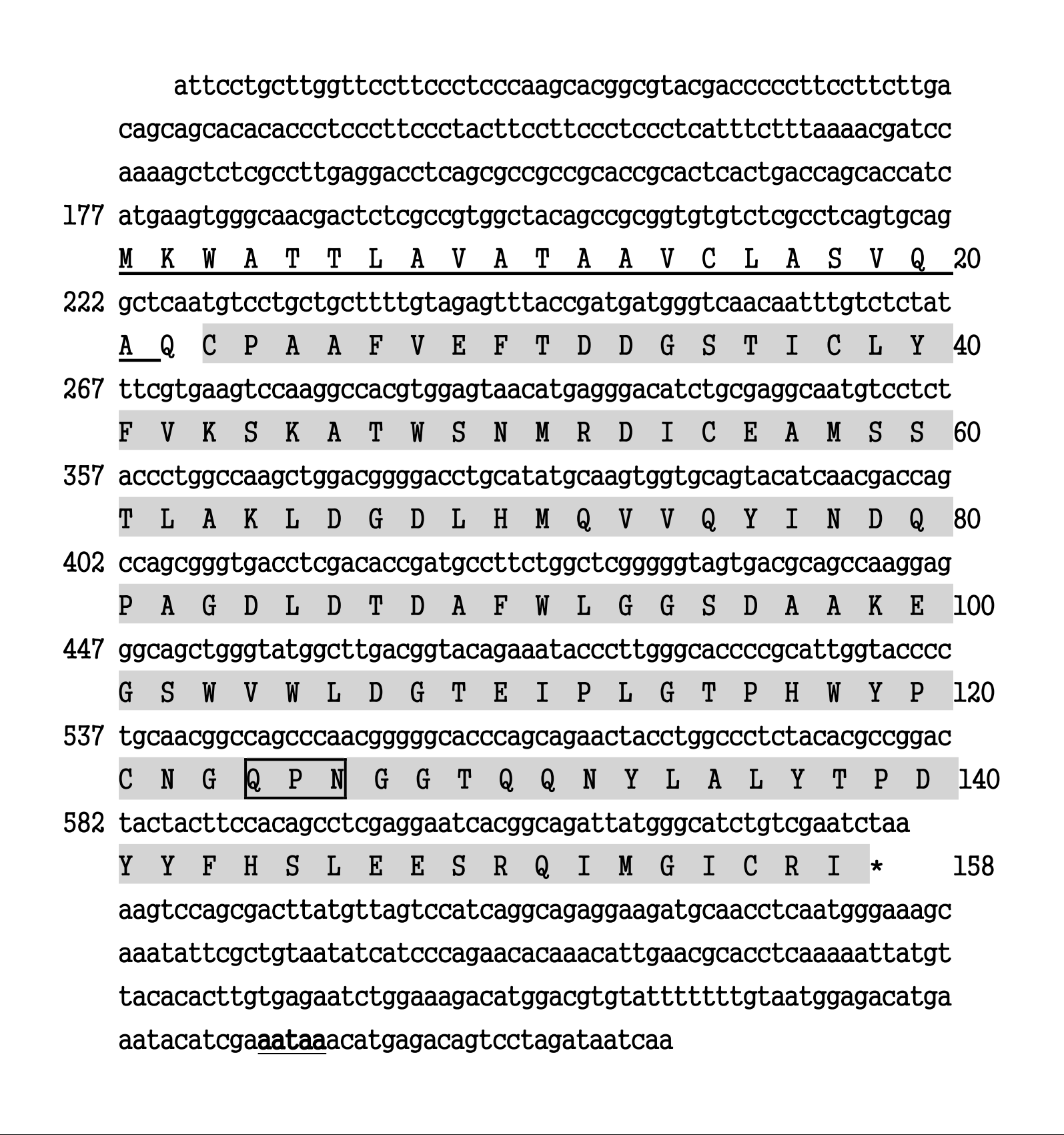

Supplement: Figure S1 — Nucleotide and deduced amino acid sequences of EsLecF . The nucleotide sequence is numbered from the first base at the 5′ end. The first methionine (M) is numbered as the first deduced amino acid. The bold underline indicates the location of the signal peptide (1–21 aa). The CRD is shaded (23–158 aa). The functional motif of “QPN” is bolded. (TIF) [file pone.0076132.s001.tif]

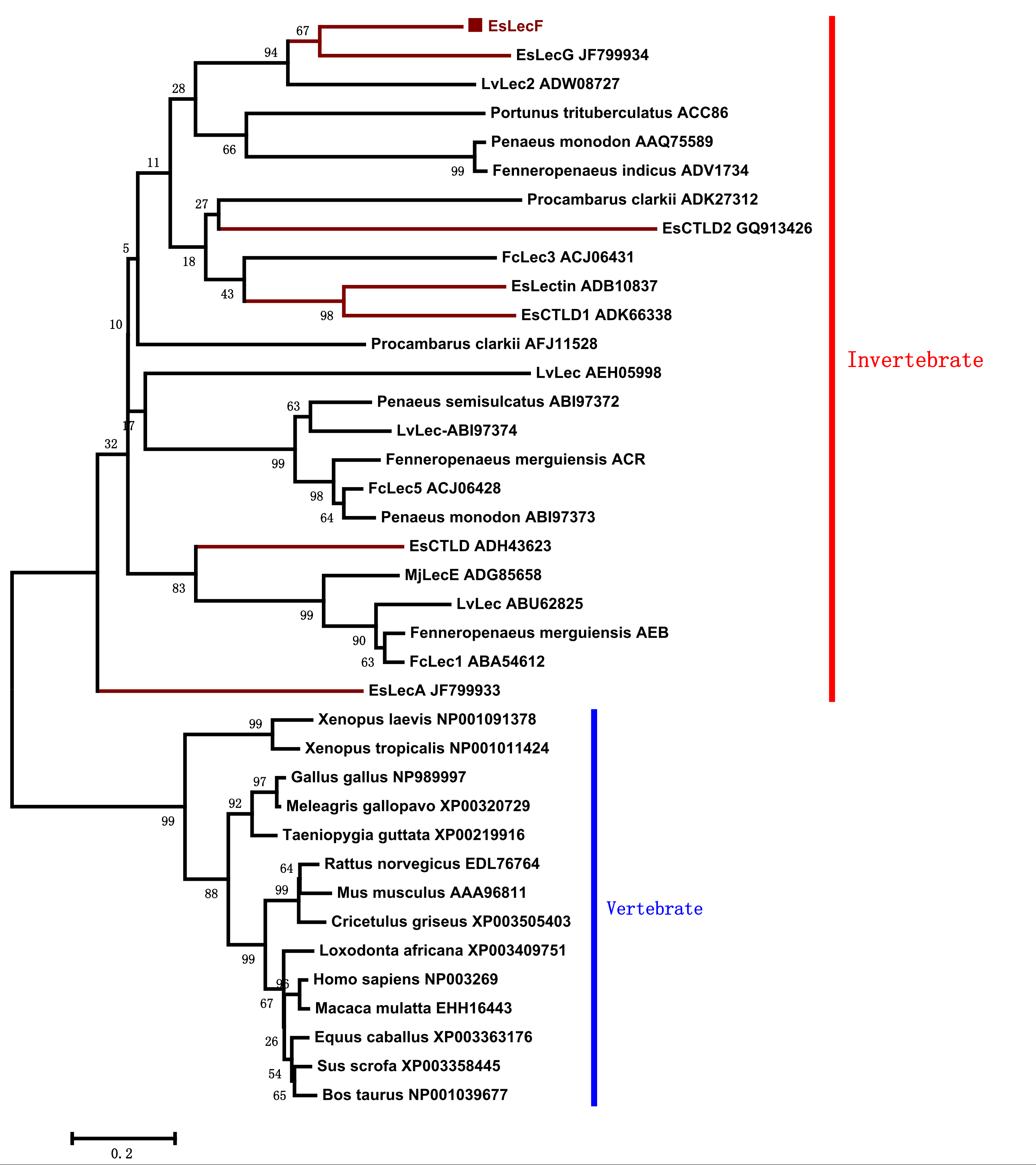

Supplement: Figure S2 — Unrooted maximum likelihood phylogenetic tree of EsLecF (labeled with a square). Amino acid sequences of C-type lectins obtained from a BlastP homology search show high similarities. The branches of E. sinensis lectins are marked in deep red color. (TIF) [file pone.0076132.s002.tif]
